# Partial Thyroidectomy

**Published:** 1912-07

**Authors:** 


### Abstracts and Selections.

### Partial Thyroidectomy.

Dunhill is in favor of operating under local anesthesia, because patients are safe, distress is negligible, the recurrent laryngeal nerve may be guarded, and post-operative vomiting does not occur. The doctor uses 7 oz. of 2 to 1000 novocain, well infiltrating all the front of the neck. In regard to the question as to how much gland tissue has to be removed he says: "We have to remove enough to cure the disease, and we must leave enough for physiological purposes, and all the judgment and experience which one has are called for. In young patients, and those not very bad, the larger lobe should be enough, with any mid-lobe or isthmus. In older people, and those in whom the disease is very bad, the removal of one lobe will practically not do any good at all; one lobe and half the other must be removed. If the gland has been big and active, even then the remaining portion may be too large. One has to remember that more can always be removed, but it can not be put back again easily. One must remove enough, but one must never be hustled into removing too much in order to make a 'complete job' at once. Although I now rarely have to operate more than once, it will occasionally happen that more has to be removed in order to completely cure the patient, and in three cases I have operated three times."—*Lancet Clinic.*

---

A Simple Means of Removing Plaster Apparatus.—In spite of the use of special instruments, the removal of apparatus containing plaster of Paris is often troublesome, and in the case of a recent fracture may cause injury. Methods of softening the plaster by water, either alone or with the addition of salt, are rarely successful, as the apparatus becomes coated with a layer of grease which prevents their action. The writer has obtained satisfactory results by moistening the line of section with vinegar applied on a tampon of wool. After a minute the plaster will be found completely softened so that it may be easily divided with a pocketknife or ordinary scissors—a procedure easy for the surgeon and painless for the patient. By this method a plaster cast for a fracture of the femur, consisting of eight turns of bandage, may be removed in about a minute and a half.—*Stransky* (*La Samaine Medicale*)—*Southern Practitioner.*

